# The Antecedents and Outcomes of Public Service Motivation: A Meta-Analysis Using the Job Demands–Resources Model

**DOI:** 10.3390/bs14100861

**Published:** 2024-09-24

**Authors:** Hanyu Tang, Shiwen An, Luoyi Zhang, Yun Xiao, Xia Li

**Affiliations:** 1 School of Sociology, Nankai University, Tianjin 300350, China; 2120232620@mail.nankai.edu.cn; 2 Zhou Enlai School of Government, Nankai University, Tianjin 300350, China; 2120232623@mail.nankai.edu.cn; 3 Faculty of Humanities and Social Sciences, City University of Macau, Macao 999078, China; h22091100245@cityu.edu.mo; 4 General English Department of School of Foreign Studies, Nankai University, Tianjin 300350, China; xiaoyun@nku.edu.cn

**Keywords:** public service motivation, JD-R model, meta-analysis

## Abstract

Understanding what drives public service motivation and its impacts is crucial for improving public sector performance. This meta-analysis synthesized the antecedents and outcomes of public service motivation based on the job demands–resources (JD-R) model. Incorporating 177 studies and 179 independent samples, with a total of 319 effect sizes, the results indicated the following: (1) Job resources and personal resources were positively related to public service motivation, while hindrance demands were negatively related to it. (2) Public service motivation had a positive relationship with overall job attitudes, job satisfaction, organizational commitment, and work engagement. It also had a positive relationship with overall job performance, in-role performance, and extra-role performance. (3) In terms of the antecedents, individualism/collectivism moderated the relationship between job resources and public service motivation, as well as the relationship between hindrance demands and public service motivation. As for the outcomes, individualism/collectivism moderated the relationship between public service motivation and both job attitudes and job performance. This study contributes to a comprehensive understanding of the antecedents and outcomes of public service motivation, offering valuable insights for future research and serving as a reference for theory development and practical application.

## 1. Introduction

Public service motivation (PSM) is a concept that has garnered significant attention in public administration and organizational behavior. It is defined as “an individual’s predisposition to respond to motives grounded primarily or uniquely in public institutions” [1]. Perry (1996) suggested that PSM primarily consists of four dimensions: attraction to public policy making (APM), commitment to the public interest (CPI), compassion (COM), and self-sacrifice (SS) [2]. Other scholars proposed that PSM may encompass one or three dimensions [3,4]. Despite these varying perspectives, the dimensions of PSM collectively reflect an individual’s propensity to serve others for the enhancement of society and the community.

Current research on PSM primarily focuses on its antecedents and outcomes. Among them, the research on antecedents predominantly explores job and personal characteristics. Studies have demonstrated the significant role of social support in fostering PSM [5,6], the effectiveness of goal clarity in positively predicting PSM [7], and the positive association of self-efficacy with PSM [8]. Moreover, a previous meta-analysis found that demographic characteristics, such as age, have a minor relationship with PSM [9]. In terms of the outcomes, the research mainly focuses on the beneficial consequences of PSM, including higher levels of work engagement and satisfaction [10,11]. It is found that individuals with a high level of PSM are more inclined to exhibit positive behaviors in the workplace, such as citizenship behaviors and proactive behaviors [11,12,13].

While substantial literature exists on the antecedents and outcomes of PSM, several theoretical issues still need to be addressed to fully understand the role of perceptions of PSM. First, existing research is relatively fragmented, lacking the integration of the findings into a cohesive theoretical framework. The key job factors and personal factors influencing PSM also remain to be fully explored. Second, although there have been meta-analyses attempting to integrate the literature on PSM, they have primarily concentrated on a single aspect of PSM or explored a limited range of its correlates, such as job satisfaction [14], work engagement [10], task performance [15], or demographic characteristics [9]. Moreover, inconsistencies persist in the relationship between PSM and other variables. For example, some studies indicate that PSM has a negative relationship with turnover intention [13,16], while others indicate no significant relationship between the two [12,17]. Therefore, a more comprehensive meta-analysis is needed to examine the relationship between PSM and its antecedents, as well as that between PSM and its outcomes, thoroughly.

Meta-analysis can correct sampling errors and measurement errors in research findings, enhancing the accuracy of estimated relationships between specific variables [18]. It can also identify moderating variables and explore potential boundary conditions for variable relationships so as to clarify differences across studies. Given this methodological strength, we aim to integrate the antecedents and outcomes of PSM through a meta-analysis. First, we conduct a comprehensive quantitative synthesis of the antecedents and outcomes of PSM based on the job demands–resources (JD-R) model to draw broader and more precise conclusions. Second, we identify the key factors influencing PSM by comparing the magnitudes of various antecedents. Third, given the significant impact of cultural differences on PSM [10], this study will also examine the moderating role of cultural values (e.g., individualism/collectivism) to provide insights for future cross-cultural research. Compared with previous meta-analyses, this study involved a broader range of literature in the exploration of the relationship between PSM and broader outcomes (e.g., turnover intention, extra-role performance). Most importantly, we have employed a meta-analysis to comprehensively examine, for the first time, the impact of job and personal characteristics on PSM. This study is expected to offer insights for future research and serve as a reference for both theory development and practical application.

## 2. Theoretical Basis and Model Construction

The job demands–resources (JD-R) model is a widely recognized framework in organizational psychology that helps explain how various job and personal characteristics influence employee motivation, strain, and work outcomes [19]. This study posits that the JD-R model provides an effective theoretical framework for understanding PSM.

First, the JD-R model categorizes job and personal characteristics that influence work motivation into three main factors: job resources, personal resources and job demands. Job and personal resources serve as catalysts that enhance individual motivation and contribute to positive work outcomes. Job demands are further divided into challenge demands and hindrance demands [20]. Challenge demands, such as high workload or time pressure, can stimulate individual motivation and improve performance, while hindrance demands, such as role ambiguity or red tape, can diminish motivation and hinder performance [20]. Previous research suggests that PSM is a form of autonomous work motivation [21]. Therefore, this framework can also be applied to understanding the antecedents of PSM.

Second, the JD-R model proposes that resources and demands impact work outcomes through two distinct pathways: the health impairment pathway and the motivational pathway [20]. Within this framework, PSM functions as a motivational pathway, mediating the relationships between resources, demands, and work outcomes (e.g., job attitudes and job performance). For example, Caillier has demonstrated that PSM could positively mediate the relationship between job resources and organizational commitment, as well as the relationship between job resources and extra-role performance [7]. Another study has shown that PSM could mediate the effects of challenge demands and hindrance demands on presenteeism [22].

Therefore, the JD-R model could effectively integrate the antecedents and outcomes of PSM into a cohesive and comprehensive framework. Given the model, this study divided the PSM antecedents into three broad categories: job resources, personal resources, and job demands. Additionally, the outcomes were classified into job attitudes and job performance.

### 2.1. Antecedents of PSM

In the JD-R model, job resources are the physical, psychological, social, or organizational aspects of the job that assist individuals to achieve work-related goals and promote personal growth, learning, and development [20]. Examples of job resources include social support, job autonomy, career opportunities, organizational justice, and goal clarity [19,23,24]. According to self-determination theory, the satisfaction of basic psychological needs can promote work motivation [25]. Previous research has shown that job resources can facilitate the satisfaction of basic psychological needs (e.g., competence needs) [26], thereby enhancing PSM [21]. Additionally, an empirical study has also concluded that job resources, such as social support, can help alleviate work stress, which in turn facilitates the stimulation of individuals’ PSM [5].

Personal resources encompass an individual’s perception of their ability to control and influence their environment, such as self-efficacy and resilience [24]. As personal resources increase, individuals tend to have a more positive view of themselves. Several studies have shown that individuals’ positive self-regard (e.g., self-efficacy, resilience) can promote their prosocial tendencies [27,28,29]. PSM is defined as a type of altruistic psychological inclination [1]. Thus, it can be supposed that personal resources will play an important role in promoting PSM.

Therefore, this study hypothesizes:

**H1.**  *Job resources and personal resources are positively related to PSM.*

Challenge demands involve the pressure or demands associated with job tasks that are perceived as opportunities for growth, skill development, and motivation (e.g., workload, time pressure) [30]. Hindrance demands refer to the pressures or demands that deplete psychological resources, hinder individual capability development and goal achievement (e.g., role ambiguity, red tape) [31]. Evidence suggests that challenge demands can promote work motivation, while hindrance demands reduce work motivation [31]. Additionally, based on affective event theory [32], challenge demands can increase positive affect [33,34], thereby promoting individuals’ prosocial behaviors [30,35]. In contrast, hindrance demands would trigger negative affect [30], consequently diminishing individuals’ prosocial behavior [36]. PSM can be considered as a form of prosocial orientation, as well as work motivation [21]. Therefore, this study supposes:

**H2.**  *Challenge demands (H2a) are positively related to PSM. Hindrance demands (H2b) are negatively related to PSM.*

### 2.2. Outcomes of PSM

Job attitudes are individuals’ subjective evaluations of their job, encompassing their emotions, beliefs, and attachments towards their job, such as job satisfaction, organizational commitment, work engagement, and turnover intention [37]. Job performance refers to an employee’s behaviors that can help to achieve organizational goals [38]. Job performance can be categorized as in-role performance and extra-role performance. The former is behavior that aligns with formal job descriptions (e.g., task performance, productivity), while the latter encompasses actions that go beyond formal job requirements (e.g., OCBs, proactive behaviors) [39].

In the JD-R model, work motivation is the proximal variable of work outcomes [19]. This study proposes PSM would have a positive impact on job attitudes and performance. Individuals with higher levels of PSM may find it easier to realize their prosocial needs in public organizations, thereby they are more satisfied with their jobs and have higher levels of work engagement [40,41]. From the perspective of person–organization fit, researchers suggest that PSM can help individuals align with the values of public organizations, thereby enhancing job satisfaction and organizational commitment [42]. Gan et al. also found that PSM can reduce turnover intention by increasing job satisfaction and organizational commitment [16].

Individuals with positive job attitudes (e.g., job satisfaction, organizational commitment) are more likely to exhibit enhanced in-role and extra-role performance [43]. Consequently, PSM also has a positive influence on in-role performance and extra-role performance. Empirical evidence supports this assertion. For example, Schwarz et al. identified a positive correlation between PSM and task performance [44]. Campbell et al. found that PSM positively predicts change-oriented OCB [45].

Therefore, this study proposes:

**H3.**  *PSM is positively related to overall job attitudes and job performance*.

### 2.3. Culture Values as Moderators

Rattrie et al. emphasized the significance of cultural values as pivotal boundary conditions within the JD-R model [46]. Specifically, they found that cultural values (e.g., individualism/collectivism, tightness/looseness) can moderate the relationship between job demands and engagement, as well as that between job resources and engagement [46]. Culture-fit theory also suggests that national culture, as a high-level social context, can impose constraints on organizational policies and practices, thereby influencing the attitudes and behaviors of individuals within the organizations [39,47]. Drawing from these perspectives, this study supposes that the relationship between PSM and its antecedents and outcomes may be moderated by the corresponding cultural values.

Individualism/collectivism is a crucial dimension in national culture [48]. It reflects how much a society prioritizes individual goals and achievements versus collective goals and group harmony, which is likely to have a particular impact on PSM [9,49]. Individualistic cultures place a high value on personal freedom, independence, and individual interests, whereas collectivist cultures emphasize group harmony, cooperation, and collective interests [49]. Kim found that in cultures exhibiting a stronger inclination towards individualism, the level of individual PSM tends to diminish [50].

According to the conservation of resources (COR) theory, an individual’s resource investment is influenced by cultural values [51]. Compared to individualistic cultures, individuals in collectivist cultures are more inclined to allocate resources to uphold group benefits [51]. For example, Rockstuhl et al. found that perceived organizational support, a type of social support in organizations, has a stronger relationship with organizational citizenship behavior in collectivist cultures [52]. It is expected that the association between job/personal resources and PSM is stronger in collectivist cultures compared to individualistic cultures. Based on these insights, this study hypothesizes:

**H4.**  *Individualism/collectivism moderates the relationship between resources (both job and personal) and PSM, with stronger correlations observed in collectivist cultures.*

Collectivist cultures prioritize access to training, favorable working conditions, and the application of one’s skills, whereas individualistic cultures value jobs that offer ample personal time and the autonomy to execute tasks according to individual preferences [48]. Therefore, individuals in individualistic cultures, when facing certain challenge demands such as time pressure and workload, are more likely to perceive them as obstacles, thus diminishing the positive effects of challenge demands. In contrast, individuals in collectivist cultures are more likely to view these challenge demands as opportunities to utilize their skills, thereby enhancing the positive effects of challenge demands. Empirical studies support this distinction: in collectivistic cultures (e.g., China), challenge demands are significantly positively correlated with work engagement [53], whereas in individualistic cultures (e.g., USA), the correlation is not significant [54].

Hindrance demands are more closely related to work context issues (e.g., organizational politics, job insecurity) [34], and collectivist cultures place a greater emphasis on good working conditions [55]. Thus, hindrance demands may have a stronger negative impact on individuals in collectivist cultures. For example, Probst and Lawler found that individuals in collectivist cultures, compared to those in individualistic cultures, report lower job satisfaction, higher turnover intentions, and increased withdrawal behaviors when confronted with hindrance demands like job insecurity [56].

Therefore, it is hypothesized:

**H5.**  *Individualism/Collectivism moderates the relationship between demands (both challenge and hindrance) and PSM, with a stronger correlation observed in collectivist cultures.*

From a culture-fit perspective [39], self-sacrifice and dedication to public interests resonate more deeply within collectivist cultures. Consequently, PSM is likely to exert a more substantial positive influence on job attitudes and behaviors in these cultures than in individualistic cultures. Supporting this notion, research by Ding and Wang demonstrates a stronger correlation between PSM and work engagement in collectivist cultures [10]. This leads us to the following hypothesis:

**H6.**  *Individualism/Collectivism moderates the relationship between PSM and job outcomes (both attitudes and performance), with a stronger correlation observed in collectivist cultures.*

**Figure 1:**
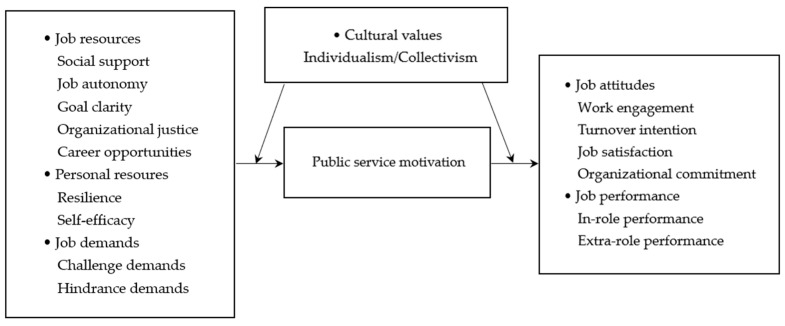
The proposed model of meta-analysis.

The research model is shown in Figure 1.

## 3. Materials and Methods

### 3.1. Literature Search and Screening

To ensure the quality of the literature included in this study, we focused exclusively on papers published in core journals (CSSCI, SSCI, SCI). Our literature search involved both Chinese and English databases, specifically CNKI (limited to CSSCI) and Web of Science (limited to Core Collection), as they are the largest comprehensive databases for Chinese and English literature, respectively, ensuring the inclusion of relevant studies across various fields. Additionally, we selected Wiley (Hoboken, NJ, USA), Springer (Berlin/Heidelberg, Germany), SAGE (Newcastle upon Tyne, UK), Elsevier (Amsterdam, The Netherlands, ScienceDirect), Emerald (Bingley, UK), and Taylor & Francis (Abingdon, UK) because they are prominent publishers in the social sciences, which helps to prevent omissions and ensures comprehensive coverage. This approach enabled us to include the most relevant, high-quality, and peer-reviewed studies essential for a robust meta-analysis. The following keywords were used:public service motivation, public service-oriented motivation, and public sector motivation. The search, encompassing literature published up to April 2024, was comprehensive and current.

**Figure 2:**
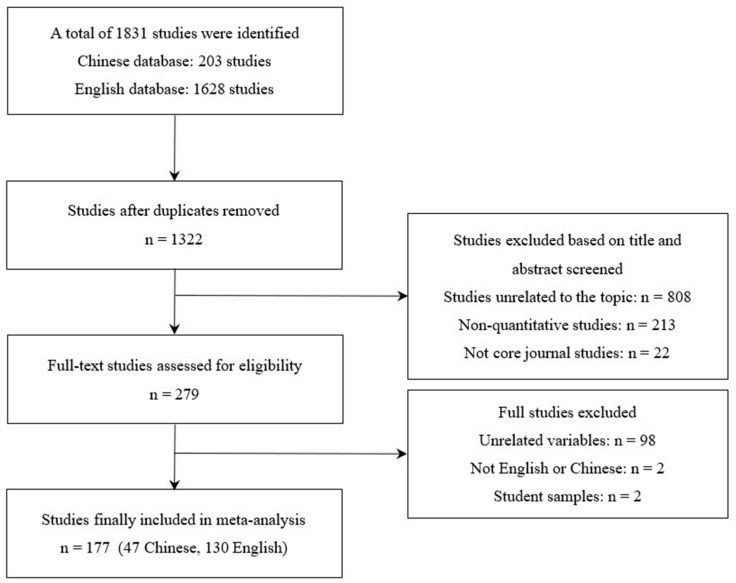
PRISMA literature screening diagram.

To ensure thoroughness and avoid missing any relevant studies, the first and second authors conducted the literature screening process and chose the final studies. The specific steps were as follows: Firstly, the first author imported the retrieved studies into Endnote and removed the duplicates, resulting in 1322 studies. Secondly, the first and second authors manually and independently screened the titles and abstracts based on the following criteria: (1) studies related to the topic (checked whether the titles or abstracts mentioned antecedents or outcomes related to PSM); (2) studies that were empirical and quantitative (e.g., checked whether the abstract described the research design, data collection methods, or statistical analysis results); (3) studies published in core journals (SSCI/SCI/CSSCI). This process narrowed the selection to 279 studies. Subsequently, both authors read the full texts of these studies and applied the following criteria: (1) studies needed to include related variables (job resources/personal resources/job demands/job attitudes/job performance); (2) studies published in either Chinese or English; (3) studies needed to report sample sizes and correlations, or statistics convertible to correlations (e.g., *F*, *t*), between PSM and other variables; (4) given the focus on workplace dynamics, studies had to involve non-student samples. This final screening yielded 177 studies, including 179 independent samples and 319 effect sizes. The literature screening process is shown in Figure 2.

### 3.2. Coding Procedure

The first and second authors independently coded each study that met the inclusion criteria. Specifically, they recorded the journal name, title, first author, publication year, sample size, *r* (correlation coefficient indicating the relationship between PSM and other variables), reliability, and country/region. When a study reported correlations for different dimensions of the same variable, we used Hunter–Schmidt’s method to combine them [18]. When a study reported results obtained from multiple independent samples, each sample was coded separately. The turnover intention was adjusted and integrated into broader job attitude categories through reverse coding. To analyze cultural differences, we used the median split method to categorize Hofstede’s cultural dimensions into high and low groups [57]. Specifically, according to Hofstede’s cultural dimensions survey scores [48], the countries or regions with scores above 50 on the individualism index were classified into the individualism group, while those with scores below 50 were classified into the collectivism group. The coding process achieved 89.4% consistency. Any discrepancies in the coding were discussed and resolved to reach the final results.

### 3.3. Meta-Analytic Procedure

This study employed the meta-analysis approach developed by Hunter and Schmidt using the psychmeta package in R 4.2.2 [18]. This method calculates mean correlations using a random-effects model, which accounts for the variability in true effects across studies, enhancing the generalizability of the results. For the main effects analysis, this study computed the meta-analytic correlation corrected for reliability (Cronbach’s α) in both the PSM and its antecedents or outcomes. Since not all studies reported reliability coefficients, the artifact-distribution method was employed to obtain the corrected correlations. Specifically, the uncorrected mean corrections were first meta-analyzed, simply weighted by sample size. The results of this “barebones” meta-analysis were then corrected using the means and variances of the observed reliability distributions [58].

Following the suggestions by Hunter and Schmidt [18], this study provided the sample size-weighted mean correlation ($\bar{r}$) and reliability-corrected mean correlation ($\bar{\rho }$), along with the true score standard deviation (*SD_ρ_*), the 95% confidence interval (95% CI), and the 80% credibility interval (80% CV) of $\bar{\rho }$. Hypotheses are accepted when the 95% CI of $\bar{\rho }$ does not include zero, indicating a statistically significant relationship. Heterogeneity in meta-analysis refers to the degree of variation in effect sizes across different studies. In this study, heterogeneity was assessed based on the percentage of variance explained by statistical errors (%VAR). If %VAR is less than 75%, significant heterogeneity between studies exists, suggesting the presence of moderator variables [18].

Moderator analyses were performed using the subgroup analysis and one-way analysis of variance (ANOVA). The ANOVA was conducted to compare the reliability-corrected mean correlations across different subgroups, and a significant *F*-value indicates a moderating effect, thereby supporting the hypotheses.

Publication bias refers to the distortion in results that occurs when the published studies are not representative of the overall body of research. Fail-safe *N* and Begg’s rank correlation test were used to estimate the extent of the publication bias in our meta-analysis. If the fail-safe *N* is more than 5*K* + 10 (*K* = number of included independent samples) and Begg’s rank correlation is non-significant, it suggests that the result of meta-analysis does not suffer from serious publication bias.

## 4. Results

### 4.1. Main Effects

Table 1 shows the meta-analysis results regarding the relationship between PSM and its antecedents. The effect sizes were interpreted according to Cohen’s guidelines (*r* ≥ 0.1 small, *r* ≥ 0.3 medium, and *r* ≥ 0.5 large) [59], allowing us to differentiate the magnitude of correlation coefficients. Job resources showed a medium and positive relationship with PSM ($\bar{\rho }$ = 0.30, 95% CI [0.24, 0.36]). Specifically, social support ($\bar{\rho }$ = 0.38, 95% CI [0.26, 0.51]), job autonomy ($\bar{\rho }$ = 0.19, 95% CI [0.12, 0.26]), goal clarity ($\bar{\rho }$ = 0.35, 95% CI [0.20, 0.50]), and organizational justice ($\bar{\rho }$ = 0.29, 95% CI [0.15, 0.44]) were positively related to PSM. However, career opportunities were unrelated to PSM ($\bar{\rho }$ = 0.16, 95% CI [−0.08, 0.41]). Personal resources also exhibited a medium and positive association with PSM ($\bar{\rho }$ = 0.43, 95% CI [0.30, 0.57]). Both resilience ($\bar{\rho }$ = 0.50, 95% CI [0.27, 0.73]) and self-efficacy ($\bar{\rho }$ = 0.34, 95% CI [0.12, 0.55]) demonstrated a positive association with PSM. Conversely, challenge demands were not related to PSM ($\bar{\rho }$ = −0.02, 95% CI [−0.10, 0.06]), while hindrance demands were negatively related to PSM ($\bar{\rho }$ = −0.15, 95% CI [−0.24, −0.06]). Therefore, H1 and H2b are supported, yet H2a was rejected.

Table 2 presents the meta-analysis results between PSM and its outcomes. PSM exhibited a significant association with overall job attitudes ($\bar{\rho }$ = 0.37, 95% CI [0.33, 0.41]). Specifically, it showed a positive relationship with work engagement ($\bar{\rho }$ = 0.61, 95% CI [0.56, 0.66]), job satisfaction ($\bar{\rho }$ = 0.31, 95% CI [0.26, 0.37]), and organizational commitment ($\bar{\rho }$ = 0.45, 95% CI [0.37, 0.52]). However, PSM was not related to turnover intention ($\bar{\rho }$ = −0.07, 95% CI [−0.14, 0.01]). PSM had a positive relationship with overall job performance ($\bar{\rho }$ = 0.51, 95% CI [0.46, 0.56]), in-role performance ($\bar{\rho }$ = 0.43, 95% CI [0.33, 0.52]) and extra-role performance ($\bar{\rho }$ = 0.55, 95% CI [0.50, 0.60]). Thus, H3 was supported.

### 4.2. Moderating Effects

Table 3 presents the moderation analysis results, highlighting the effect of cultural values on the relationship between PSM and related variables. There was a significant cultural difference regarding the relationship between job resources and PSM (*F* (1, 46.02) = 55.90, *p* < 0.001). Specifically, the correlation between job resources and PSM in collectivist cultures ($\bar{\rho }$ = 0.45, 95% CI [0.39, 0.51]) was significantly stronger than that in individualistic cultures ($\bar{\rho }$ = 0.15, 95% CI [0.10, 0.20]). No significant moderating effect of individualism/collectivism was observed for the relationship between personal resources and PSM (*F* (1, 4.71) = 0.08, *p* = 0.79). A significant relationship between personal resources and PSM was found in collectivist cultures ($\bar{\rho }$ = 0.47, 95% CI [0.29, 0.65]). However, personal resources were not related to PSM in individualistic cultures ($\bar{\rho }$ = 0.36, 95% CI [−0.06, 0.78]). Additionally, individualism/collectivism did not moderate the relationship between challenge demands and PSM (*F* (1, 1.14) = 0.00, *p* = 1.00). The correlation between challenge demands and PSM was not significant in both individualistic ($\bar{\rho }$ = −0.02, 95% CI [−1.97, 1.93]) and collectivist ($\bar{\rho }$ = −0.02, 95% CI [−0.10, 0.07]) cultures. There was a significant cultural difference in the relationship between hindrance demands and PSM (*F* (1, 19.96) = 12.12, *p* = 0.002). In collectivist cultures, the negative correlation between hindrance demands and PSM ($\bar{\rho }$ = −0.26, 95% CI [−0.39, −0.13]) was stronger compared to that in individualistic cultures ($\bar{\rho }$ = −0.04, 95% CI [−0.06, −0.01]). Consistent with our hypothesis, a significant cultural difference was observed in the relationships between PSM and job attitudes (*F* (1, 95.31) = 26.28, *p* < 0.001), as well as job performance (*F* (1, 32.82) = 30.66, *p* < 0.001). In collectivist cultures, the correlation between PSM and job attitudes ($\bar{\rho }$ = 0.49, 95% CI [0.44, 0.54]), as well as job performance ($\bar{\rho }$ = 0.59, 95% CI [0.54, 0.64]), was stronger compared to that in individualistic cultures ($\bar{\rho }$ = 0.28, 95% CI [0.22, 0.34]; $\bar{\rho }$ = 0.34, 95% CI [0.26, 0.42]). Therefore, H6 was supported while H4 and H5 were partially supported.

### 4.3. Publication Bias Tests

The results of the publication bias tests are shown in Table 4. The fail-safe *N* statistic, which indicates the number of unpublished studies required to nullify the significant meta-analysis results, was reported only for significant correlations. Therefore, we reported the fail-safe *N* only when the correlation was significant. It can be observed that the fail-safe *N* between PSM and each variable exceeded 5*K* + 10, and the Begg rank correlations were also not significant (*p* > 0.05), suggesting that our meta-analysis does not suffer from a substantial publication bias.

## 5. Discussion

The JD-R theory indicates that resources and demands can impact individual work motivation, thereby influencing work-related outcomes. This research confirmed the utility of the JD-R model in understanding PSM, while also examining the moderating effect of cultural values. Out of the seven hypotheses tested, four hypotheses were fully supported (H1, H2b, H3, H6), two were partially supported (H4, H5), and one was not supported (H2a). These results reinforce the applicability of the JD-R model to PSM and highlight the critical role of cultural values in moderating these relationships.

### 5.1. Main Effects of Antecedents

The study found that overall job resources were moderately related to PSM ($\bar{\rho }$ = 0.3). This suggests that job resources can contribute to improving PSM. According to the JD-R model, job resources provide employees with the psychological and physical support that fosters personal growth and reduces job stress [19]. When employees feel more competent and capable, they are more likely to be motivated and committed to public service. Among the five types of job resources considered in this study, social support exhibited the strongest correlation with PSM ($\bar{\rho }$ = 0.38). Social support can provide employees with instrumental and emotional support, which helps them cope with stress and consequently enhances PSM [5]. Furthermore, from a social exchange perspective, social support (e.g., organizational support) can stimulate employees’ sense of reciprocity towards the organization or society [52], thereby boosting their PSM [6]. Thus, the combined effects of stress alleviation and a sense of reciprocity result in the strongest correlation between social support and PSM. However, our meta-analysis indicated that career opportunities had a non-significant relationship with PSM. One possible explanation is that individuals attracted to public service may prioritize factors such as making a positive impact on society, serving the community, or aligning with personal values over traditional career progression [60]. Moreover, career opportunities typically align with extrinsic motivation [61], while PSM is more related to intrinsic motivation [21]. Previous research indicates that extrinsic motivation can diminish intrinsic motivation [62]. Consequently, this might also result in a non-significant correlation between career opportunities and PSM.

The study also found that overall personal resources exhibited a positive correlation with PSM ($\bar{\rho }$ = 0.43). In the JD-R model, personal resources function similarly to job resources. Individuals with strong personal resources are more likely to believe in positive outcomes and their ability to handle unforeseen challenges [19]. This sense of efficacy, in turn, increases their willingness to engage in public service. The results showed that personal resources exhibited the strongest correlation with PSM among the three types of antecedents. This indicates that personal resources play a critical role in enhancing PSM. This finding is consistent with previous studies, such as those by Mazzetti et al., which demonstrate that personal resources have a greater influence on work engagement compared to job and social resources [24]. Personal resources are intrinsic, stable traits tied to an individual’s core beliefs and self-awareness, whereas job resources (e.g., social support, job autonomy) are more influenced by external factors, such as leadership styles [63]. Due to their higher stability, personal resources have more significant and long-term impacts on individuals [64]. Therefore, the impact of personal resources on PSM is stronger than that of job resources. Moreover, within the category of personal resources, resilience showed a notably stronger positive correlation with PSM ($\bar{\rho }$ = 0.50) compared to self-efficacy ($\bar{\rho }$ = 0.34). In the public service domain, individuals often encounter higher levels of occupational stress [65]. Resilience may have a stronger coping effect on occupational stress compared to self-efficacy [66,67]. Empirical research also supports this perspective. For example, a study on nurses in Chinese public hospitals indicated that resilience has a greater preventive effect on nurse burnout compared to self-efficacy [68]. Therefore, resilience is more beneficial for individuals in the public sector to cope with stress, thereby exerting a stronger influence on PSM.

In terms of job demands, the relationship between challenge demands and PSM was not statistically significant. Further moderation effect analysis suggests that this relationship remained non-significant across various cultures. This result does not support the challenge–hindrance stress model (CHM) proposed by Cavanaugh et al., which suggests that challenge stressors/demands have a positive impact on employees, while hindrance stressors/demands have a negative impact [69]. A possible explanation can be found in explicit monitoring theory, which suggests that when individuals experience performance-related stress and strive to excel, they become more self-focused [70]. This increased self-focus enhances task absorption and concentration but may also lead to a neglect of interpersonal cues and reduced sensitivity to others [71]. As a result, while challenge demands/stressors may enhance work motivation, they might not be positively associated with helping behaviors [72,73]. Since PSM encompasses both work motivation and altruistic tendencies, the differing effects of challenge demands on these two aspects could explain the non-significant relationship between challenge demands and PSM. Hindrance demands showed a weak negative correlation with PSM ($\bar{\rho }$ = −0.15). This indicates that hindrance demands may negatively impact PSM. These demands impose constraints that obstruct goal achievement and can negatively impact overall motivation [19,20]. Consequently, high hindrance demands may diminish individuals’ willingness to engage in public service. However, this correlation is considerably weaker than that between job/personal resources and PSM. This could be attributed to the JD-R model’s proposition that job demands indirectly influence work motivation through burnout, while job/personal resources can directly impact it [19]. Thus, compared to job and personal resources, hindrance demands are more distal to PSM.

### 5.2. Main Effects of Outcomes

This meta-analysis showed that PSM had a positive relationship with overall job attitudes ($\bar{\rho }$ = 0.37), work engagement ($\bar{\rho }$ = 0.61), job satisfaction ($\bar{\rho }$ = 0.31), and organizational commitment ($\bar{\rho }$ = 0.45). However, the negative correlation between the PSM and turnover intention was not statistically significant. This indicates that while PSM is beneficial for enhancing overall job attitudes, it may not directly reduce turnover intention. The non-significant correlation between PSM and turnover intention may suggest that PSM has a paradoxical effect on turnover intention. While Gan et al. indicate that PSM can reduce turnover intention by enhancing job satisfaction and organizational commitment [16], other studies suggest that high levels of PSM may lead to work stress [74], potentially increasing turnover intention [75]. Therefore, this ultimately results in the lack of a significant correlation between PSM and turnover intention. Compared to the other three types of job attitudes, PSM had the strongest relationship with work engagement, reaching a large positive correlation ($\bar{\rho }$ = 0.61). PSM can be regarded as a type of autonomous motivation [21], which closely aligns with work engagement—a positive and fulfilling state of mind towards work characterized by vigor, dedication, and absorption [24]. A meta-analysis indicates that autonomous motivation has a stronger relationship with work engagement compared to job satisfaction and organizational commitment [76], demonstrating the large correlation between PSM and work engagement.

The study also found that PSM was positively related to overall job performance ($\bar{\rho }$ = 0.51), in-role performance ($\bar{\rho }$ = 0.43), and extra-role performance ($\bar{\rho }$ = 0.55). This result indicates that PSM can enhance job performance. Within the framework of the JD-R model, PSM can serve as a source of motivation, often energizing employees and filling them with enthusiasm [21,77]. This motivation drives them to take initiative and persist in the face of challenges, ultimately leading to improved performance. Compared to in-role performance, the correlation between PSM and extra-role performance was considerably stronger. This is attributed to the fact that extra-role performance, unlike in-role performance, prioritizes actions exceeding formal job requirements to benefit the organization and its members (e.g., OCB) [39], showing a higher propensity towards altruistic behavior. Consequently, this fosters a closer association with PSM.

### 5.3. Moderating Effects of Individualism/Collectivism

The moderating effects analysis indicated that individualism/collectivism moderated the relationships between job resources and PSM. In collectivist cultures, the correlations between job resources and PSM were found to be stronger. Collectivist cultures emphasize the importance of collective interests or goals, while individualist cultures prioritize personal interests or goals [48]. As a result, employees in collectivist cultures are more inclined to use available job resources effectively for public service.

However, this study did not find a moderating effect of individualism/collectivism on the relationship between personal resources and PSM. There is no statistically significant difference in the relationship between personal resources and PSM under collectivist versus individualistic cultures. This may be attributed to the fact that personal resources are relatively stable traits, which are less likely to be influenced by cultural factors. For example, a study involving participants from 25 countries found that individualism/collectivism has a non-significant effect on general self-efficacy [78]. Additionally, recent research has also shown that culture does not moderate the relationship between personal resources and work-related flow [79].

Furthermore, individualism/collectivism did not significantly moderate the relationship between challenge demands and PSM. The correlation between challenge demands and PSM was not significant in either individualistic or collectivist cultures. One possible explanation is that the majority of the samples in this study were from the public sector, where employees globally may experience high levels of burnout [80,81]. This burnout may diminish the positive impact of challenge demands on PSM, leading to an insignificant correlation in both cultures. Therefore, future applications of the challenge–hindrance stress model should consider both the boundary conditions of the cultures and those of the work contexts.

The analysis showed that individualism/collectivism moderates the relationship between hindrance demands and PSM, with the correlation being stronger in collectivist cultures. Individualistic cultures place a higher value on preserving individual resources, viewing them primarily as contributors to individual well-being [51]. Therefore, employees in individualistic cultures tend to have more individual resources to handle hindrance demands, which results in a weaker negative impact of hindrance demands on PSM compared to collectivist cultures. Previous study has also found that the negative impact of hindrance demands on employee innovation is weaker in individualistic cultures than in collectivist cultures [82].

Regarding work outcomes, individualism/collectivism moderated the relationship between PSM and job attitudes, as well as that between PSM and job performance. In collectivist cultures, the correlations between PSM and both job attitudes and performance were stronger than in individualistic cultures. This finding supports the culture-fit perspective [39,47], which suggests that PSM—an altruistic drive to serve others or society—aligns more closely with the values emphasized in collectivist cultures. Consequently, PSM is more likely to enhance individual job attitudes and performance in collectivist cultures.

### 5.4. Limitations and Future Research

Although this study strictly adhered to the methodological process of meta-analysis, several limitations remain. Firstly, to ensure the quality of literature, we restricted inclusion to articles published in Chinese and English core journals, excluding unpublished articles and articles in other languages, which may lead to an incomplete representation of the literature. Secondly, our investigation of moderator variables focused solely on the influence of individualism/collectivism, which may limit the generalizability of the findings across different contexts. Future research could explore additional moderator variables, such as other cultural dimensions, specific work departments, and demographic factors, to further expand the boundary conditions of PSM and related variables. Thirdly, due to the constraints of the included literature, our study considered only five types of job resources and two types of personal resources. Future research could compare a broader range of job and personal resources to assess their impact on PSM. Finally, because the literature included in this study primarily measured overall PSM, our analysis focused only on the relationship between overall PSM and the related variables. Future studies could investigate how different job and personal resources impact the various dimensions of PSM, as well as how these dimensions of PSM affect work outcomes.

## 6. Conclusions

The current study conducts a meta-analytic review to explore the antecedents and outcomes relevant to PSM. The findings suggest that PSM is influenced by job resources, personal resources, and hindrance demands. It is also associated with various job attitudes and performance outcomes. The moderation analysis reveals that cultural values, specifically individualism and collectivism, play a moderating role—they influence the relationships between job resources and PSM, hindrance demands and PSM, and PSM and work outcomes.

This study provides some theoretical implications. Firstly, it systematically elucidates the antecedents and outcomes of PSM based on the JD-R model, identifying key antecedents and outcomes. This not only deepens the understanding of PSM but also expands the application of the JD-R model in the public service domain. Secondly, this study examines the moderating effect of individualism/collectivism, extending the boundary conditions of the antecedents and outcomes of PSM, which contributes to subsequent cross-cultural research.

This study also has several practical contributions. Firstly, organizations should prioritize providing employees with ample job resources and cultivate employees’ personal resources through practices such as the following: (1) a comprehensive social support system should be established within the organization, including mutual aid mechanisms, fostering strong interpersonal relationship networks, and regularly conducting team-building activities; (2) ensuring each employee comprehensively understands the organization’s mission and objectives through regular communication and feedback, aligning their personal goals accordingly; (3) implementing a fair system of job rewards, including equitable salary structures, promotion opportunities, and resource allocation; (4) providing employee assistance program (EAP) services regularly to help employees solve personal problems, manage stress and emotions, and enhance self-efficacy and resilience. Secondly, organizations should also take corresponding measures to reduce hindrance demands. For example, organizations can streamline workflow processes to reduce unnecessary bureaucracy. Providing employees with clear role guidance and training helps them better understand their responsibilities, thereby reducing role stress. This study found that in collectivist cultures, the relationship between PSM and both variables was stronger than in individualistic cultures. This suggests that organizations in collectivist cultures should pay more attention to cultivating employees’ PSM.

## Figures and Tables

**Table 1:** Meta-analysis results for correlations between antecedents and PSM.

Variables	*K*	*N*	$\bar{r}$	$\bar{\rho }$	*SD* *_ρ_*	95% CI	80% CV	%VAR
Job resources	49	43,208	0.25	0.30	0.20	[0.24, 0.36]	[0.03, 0.57]	4.24
Social support	16	9049	0.31	0.38	0.23	[0.26, 0.51]	[0.08, 0.68]	5.05
Job autonomy	7	6740	0.16	0.19	0.07	[0.12, 0.26]	[0.10, 0.29]	27.21
Goal clarity	12	13,271	0.29	0.35	0.23	[0.20, 0.50]	[0.04, 0.66]	3.06
Organizational justice	10	9496	0.24	0.29	0.20	[0.15, 0.44]	[0.01, 0.57]	4.08
Career opportunities	4	4652	0.13	0.16	0.15	[−0.08, 0.41]	[−0.08, 0.41]	5.65
Personal resources	11	5757	0.36	0.43	0.19	[0.30, 0.57]	[0.17, 0.70]	6.58
Resilience	5	3408	0.42	0.50	0.18	[0.27, 0.73]	[0.23, 0.78]	6.44
Self-efficacy	6	2349	0.28	0.34	0.19	[0.12, 0.55]	[0.05, 0.62]	8.46
Job demands								
Challenge demands	19	19,001	−0.02	−0.02	0.16	[−0.10, 0.06]	[−0.23, 0.19]	5.06
Hindrance demands	30	40,822	−0.12	−0.15	0.23	[−0.24, −0.06]	[−0.45, 0.15]	2.15

Note: *K* = cumulative number of independent samples; *N* = cumulative number of participants; $\bar{r}$ = sample size-weighted mean correlation; $\bar{\rho }$ = reliability-corrected mean correlation (corrected for unreliability for both variables); *SD_ρ_* = true score standard deviation; 95% CI = 95% confidence interval around $\bar{\rho }$ ; 80% CV = 80% credibility intervals around $\bar{\rho }$; %VAR = percentage of variance accounted for by statistical errors.

**Table 2:** Meta-analysis results for correlations between outcomes and PSM.

Variables	*K*	*N*	$\bar{r}$	$\bar{\rho }$	*SD* *_ρ_*	95% CI	80% CV	%VAR
Job attitudes	127	147,272	0.30	0.37	0.24	[0.33, 0.41]	[0.06, 0.68]	2.82
Work engagement	21	23,565	0.50	0.61	0.10	[0.56, 0.66]	[0.47, 0.74]	18.60
Turnover intention	20	23,382	−0.05	−0.07	0.16	[−0.14, 0.01]	[−0.28, 0.15]	4.70
Job satisfaction	59	47,840	0.26	0.31	0.20	[0.26, 0.37]	[0.06, 0.57]	5.09
Org. commitment	27	52,485	0.37	0.45	0.19	[0.37, 0.52]	[0.20, 0.69]	3.96
Job performance	83	53,345	0.42	0.51	0.21	[0.46, 0.56]	[0.24, 0.78]	5.97
In-role performance	22	16,539	0.35	0.43	0.21	[0.33, 0.52]	[0.15, 0.70]	5.18
Extra-role performance	61	36,806	0.45	0.55	0.20	[0.50, 0.60]	[0.29, 0.81]	6.88

Note: Org. commitment = organizational commitment.

**Table 3:** Meta-analysis results for moderation analysis: role of individualism/collectivism.

Variables	Subgroup	*K*	*N*	$\bar{r}$	$\bar{\rho }$	*SD* *_ρ_*	95% CI	80% CV	ANOVA
Job resources	Individualism	16	21,595	0.12	0.15	0.09	[0.10, 0.20]	[0.03, 0.28]	*F* (1, 46.02) = 55.90 ***
	Collectivism	33	21,613	0.37	0.45	0.17	[0.39, 0.51]	[0.22, 0.68]	
Personal resources	Individualism	3	1964	0.30	0.36	0.16	[−0.06, 0.78]	[0.05, 0.67]	*F* (1, 4.71) = 0.08
	Collectivism	8	3793	0.39	0.47	0.21	[0.29, 0.65]	[0.17, 0.77]	
Challenge demands	Individualism	2	2107	−0.02	−0.02	0.21	[−1.97, 1.93]	[−0.68, 0.64]	*F* (1, 1.14) = 0.00
	Collectivism	17	16,894	−0.02	−0.02	0.16	[−0.10, 0.07]	[−0.23, 0.20]	
Hindrance dmands	Individualism	10	20,303	−0.03	−0.04	0.02	[−0.06, −0.01]	[−0.06, −0.01]	*F* (1, 19.96) = 12.12 **
	Collectivism	20	20,519	−0.21	−0.26	0.28	[−0.39, −0.13]	[−0.64, 0.11]	
Job attitudes	Individualism	43	83,700	0.23	0.28	0.21	[0.22, 0.34]	[0.01, 0.55]	*F* (1, 95.31) = 26.28 ***
	Collectivism	83	62,655	0.40	0.49	0.23	[0.44, 0.54]	[0.19, 0.79]	
Job performance	Individualism	18	16,389	0.28	0.34	0.15	[0.26, 0.42]	[0.14, 0.54]	*F* (1, 32.82) = 30.66 ***
	Collectivism	65	36,956	0.48	0.59	0.19	[0.54, 0.64]	[0.34, 0.83]	

Note: ANOVA = one-way analysis of variance, *** *p* < 0.001, ** *p* < 0.01.

**Table 4:** Results of publication bias tests.

Variables	*5K* + 10	*Nfs*	Begg’s Test	
			tau	*p*
Job resources	255	46,014	0.08	0.41
Social support	90	5545	−0.05	0.82
Job autonomy	45	307	0.14	0.77
Goal clarity	70	3967	0.12	0.64
Organizational justice	60	2090	−0.07	0.86
Career opportunities	30	-	0.33	0.75
Personal resources	65	3381	−0.05	0.88
Resilience	35	1400	0.20	0.82
Self-efficacy	40	425	−0.20	0.72
Job demands				
Challenge demands	105	-	0.27	0.11
Hindrance demands	160	4325	−0.16	0.20
Job attitudes	645	552,993	−0.09	0.12
Work engagement	115	32,498	−0.11	0.51
Turnover intention	110	-	0.19	0.26
Job satisfaction	305	76,194	−0.06	0.53
Org. commitment	145	62,062	−0.08	0.56
Job performance	425	316,323	−0.02	0.79
In-role performance	120	13,933	−0.01	0.96
Extra-role performance	310	197,404	0.05	0.55

Note: *Nfs* = fail-safe *N*, tau = Begg’s rank correlation.

## Data Availability

Data are available from the corresponding author upon reasonable request.
